# Impact of autoimmune risk alleles on the immune system

**DOI:** 10.1186/s13073-015-0182-y

**Published:** 2015-06-19

**Authors:** John P Ray, Nir Hacohen

**Affiliations:** Broad Institute of MIT and Harvard, Cambridge, MA 02142 USA; Center for Immunology and Inflammatory Diseases, Massachusetts General Hospital, Charlestown, MA 02129 USA; Department of Medicine, Harvard Medical School, Boston, MA 02114 USA

## Abstract

Genetic analyses of autoimmune diseases have revealed hundreds of disease-associated DNA variants, but the identity and function of the causal variants are understudied and warrant deeper mechanistic studies. Here, we highlight methods for deciphering how alleles that are associated with autoimmune disease alter the human immune system, and suggest strategies for future autoimmune genetic research.

## The challenge

The genetic mechanisms underlying human autoimmune diseases remain elusive for many reasons: the causal alleles or genes are often not known; the effects of each allele on gene function have often not been elucidated, especially as most alleles lie in less-well-understood non-coding regions; the impact of individual variants may be weak; and unknown environmental factors may be needed to provoke the effects of specific alleles. To address these deficiencies in our knowledge, studies of risk alleles will first need to define the roles of candidate variants in specific immune responses, and in time, in disease initiation, maintenance, and progression. Here, we highlight recent systematic approaches for discovering how human common genetic variants and disease-associated risk alleles alter the molecular and cellular functions of immune cells.

## How alleles affect the composition of immune cells in the blood

Three recent studies have sought to reveal the contributions of both genetics and the environment on abundance and activation markers of different cell populations in the blood and their phenotypes. Orrù et al. [1] analyzed 95 cell types and 272 immune traits in 1629 individuals using flow cytometry. They discovered 13 loci that are associated with these phenotypes, including: the CD39 locus, which affects the number of CD39+ regulatory T cells and their function; HNRPLL single nucleotide polymorphisms (SNPs) that are associated with the relative abundance of naïve and memory T cells; and several loci that are associated with multiple traits, such as variants in the HLA class I locus that affect the prevalence of multiple CD8 T-cell subsets.

In what is the largest bioresource for immune cell phenotypes, Roederer et al. [2] list ~78,000 cell phenotypes found in 669 females who are twins. From among these phenotypes, 151 of the most highly heritable or biologically interesting traits were selected for genetic association testing. From this analysis, Roederer et al. [2] found 11 genetic loci that explained ~36 % of the heritability of 19 (of the top 151) heritable immune traits. Importantly, many of these loci are associated with autoimmunity. For example, SNP rs1801274, which was previously associated with systemic lupus erythematosus, inflammatory bowel disease, and Crohn’s disease, impacts FCGR2A/CD32 receptor expression in *cis*, as well as CD27 and CD161 expression on T cells, generating a novel and concrete hypothesis for how rs1801274 alters the immune system in humans. Brodin et al. [3] also found heritable traits, including the IL12B cytokine and T-cell subtypes, in a study of 204 immune traits in twins, but concluded from their findings that most traits are in fact not heritable. These studies are the first to show systematically how the composition of the blood is controlled by common alleles, and provide hypotheses for the actions of risk alleles, which can be further studied ex vivo or mimicked in mouse models.

## The impact of common genetic variants on the activation of immune cells

Numerous studies have considered how common alleles alter cellular states, as measured by RNA or protein expression. Most studies have monitored gene expression under baseline conditions, but a few recent studies have focused on cells that are activated in response to specific environmental stimuli, such as ligation of the T-cell antigen receptor, treatment with cytokines, or treatment with pathogen components. Examples of interesting findings include: the rs12805435 variant that causes *IRF7* to have *trans* effects on interferon-inducible genes exclusively during influenza infection [4]; a SNP close to *IFNB1* that causes a delayed alteration in the response to lipopolysaccharide [5]; and an *IL2RA* SNP that affects its own expression only in activated T cells [6]. In a meta-analysis using more than 8000 non-transformed peripheral blood samples, Westra et al. [7] identified *trans* expression quantitative trait loci (eQTLs) that are relevant to autoimmune diseases, including a lupus-associated SNP in *IKZF1* that affects the expression of several relevant genes, including C1Q and five interferon-inducible genes. By teaching us how common alleles alter immune networks, these studies are generating testable hypotheses to describe human autoimmune pathogenesis that will guide research in the field and suggest new therapeutic concepts.

## Integrating datasets to hone in on probable causal alleles and their cell type-specific function

In addition to analyzing gene expression, some studies have considered how disease alleles localize within open chromatin. Maurano et al. [8] intersected autoimmune SNPs from genome-wide association studies (GWAS) with DNase I hypersensitivity sites (DHSs) from many cell types, and found that 93.2 % of the overlapping SNPs landed in known transcription factor motifs. These authors also used chromatin interaction analysis with paired-end tag sequencing technology (ChIA-PET) to find that many GWAS variants within DHSs were highly associated with the activity of distant genes, some of which were at distances of more than 500 kb from the transcription start site; these non-coding variants may underlie phenotype associations in autoimmunity. Like DHSs, ATAC-seq also finds areas of open chromatin, but has a faster workflow and reduced sample input, and we expect it to be useful for epigenomic analysis of large numbers of patients in future studies.

Farh et al. [9] aimed to pinpoint causal disease variants near genes of interest by searching for localization of SNPs in regions of H3K27ac active chromatin in many cell types, including immune cells [9]. They honed in on a set of putative causal variants and observed that variants that were associated with most autoimmune diseases were specifically enriched in active chromatin from T cells, whereas variants that were associated with lupus were most enriched in active chromatin from B cells, T cells, and monocytes. Future studies will enhance such maps by providing even more comprehensive datasets of chromatin states across immune cell types. Rare but important cell types that are likely to play important roles in autoimmunity, such as follicular helper T cells and plasmacytoid dendritic cells, should be included. These and similar studies [10] that utilize activity maps of non-coding regions not only help to pinpoint alleles and their role in specific cell types and conditions, but also specify which *cis* elements and cognate factors are likely to be impacted by variations in the DNA. These hypotheses bring us closer to a mechanistic model of how variants affect gene expression in specific cells and conditions, which can then be validated using modern perturbation tools such as CRISPR-mediated mutagenesis. By finding out which transcription factor (TF)–DNA interactions are affected by risk alleles, we will be able to predict which pathways (upstream of the TF) should be targeted for restoration of normal immune functions.

## What about the patients?

The above studies focus on healthy individuals to avoid the complex effects of disease and treatment. And yet, some variants may only show their effects in the unique context of the patient with disease, as a result of deregulated signaling, positive feedback loops, and cellular crosstalk between pathogenic cell types. Furthermore, the open chromatin of patients might reveal permissive areas where disease variants could reside. An analysis of gene expression and chromatin states in sorted cell types from patients should be performed to reveal disease-specific effects and to link these phenotypes with genotypes. To ensure that patients are controlled for the treatment of their disease, studies could piggyback on a clinical trial, during which patient treatments would typically be more uniform than those given to the general patient population. In the future, personalized therapeutics and prophylactic measures could target gene products or pathways on the basis of information derived from a patient’s cellular epigenomic landscape, as defined by open chromatin and the patient’s risk variants that localize to this open chromatin. Understanding the genetic architecture in autoimmunity is important to derive such target genes, and pathways and bioresources such as those outlined by Roederer et al. [2] could aid in the generation of new hypotheses to delineate disease mechanisms underlying autoimmunity. With improvements in tools and careful cohort selection, it is time to bring the study of common genetic variations and their effects on phenotype back to the patient, ultimately in the context of clinical trials.

## Abbreviations

DHS: DNase I hypersensitivity site
SNP: single nucleotide polymorphism
TF: transcription factor

## References

[1] Orrù V, Steri M, Sole G, Sidore C, Virdis F, Dei M (2013). Genetic variants regulating immune cell levels in health and disease. Cell.

[2] Roederer M, Quaye L, Mangino M, Beddall MH, Mahnke Y, Chattopadhyay P (2015). The genetic architecture of the human immune system: a bioresource for autoimmunity and disease pathogenesis. Cell.

[3] Brodin P, Jojic V, Gao T, Bhattacharya S, Angel CJL, Furman D (2015). Variation in the human immune system is largely driven by non-heritable influences. Cell.

[4] Lee MN, Ye C, Villani AC, Raj T, Li W, Eisenhaure TM (2014). Common genetic variants modulate pathogen-sensing responses in human dendritic cells. Science.

[5] Fairfax BP, Humburg P, Makino S, Naranbhai V, Wong D, Lau E (2014). Innate immune activity conditions the effect of regulatory variants upon monocyte gene expression. Science.

[6] Ye CJ, Feng T, Kwon H-K, Raj T, Wilson MT, Asinovski N (2014). Intersection of population variation and autoimmunity genetics in human T cell activation. Science.

[7] Westra H-J, Peters MJ, Esko T, Yaghootkar H, Schurmann C, Kettunen J (2013). Systematic identification of trans eQTLs as putative drivers of known disease associations. Nat Genet.

[8] Maurano MT, Humbert R, Rynes E, Thurman RE, Haugen E, Wang H (2012). Systematic localization of common disease-associated variation in regulatory DNA. Science.

[9] Farh KK-H, Marson A, Zhu J, Kleinewietfeld M, Housley WJ, Beik S (2015). Genetic and epigenetic fine mapping of causal autoimmune disease variants. Nature.

[10] Trynka G, Sandor C, Han B, Xu H, Stranger BE, Liu XS, Raychaudhuri S (2012). Chromatin marks identify critical cell types for fine mapping complex trait variants. Nat Genet.

